# Epidemiology and Risk Factors of Cerebral Ischemia and Ischemic Heart Diseases: Similarities and Differences

**DOI:** 10.2174/157340310791658785

**Published:** 2010-08

**Authors:** Ernest Palomeras Soler, Virgina Casado Ruiz

**Affiliations:** Hospital de Mataró, Spain

**Keywords:** Coronary heart disease, ischemic stroke, epidemiology, risk factors.

## Abstract

Cerebral ischemia and ischemic heart diseases, common entities nowadays, are the main manifestation of circulatory diseases. Cardiovascular diseases, followed by stroke, represent the leading cause of mortality worldwide. Both entities share risk factors, pathophisiology and etiologic aspects by means of a main common mechanism, atherosclerosis. However, each entity has its own particularities. Ischemic stroke shows a variety of pathogenic mechanisms not present in ischemic heart disease. An ischemic stroke increases the risk of suffering a coronary heart disease, and viceversa. The aim of this chapter is to review data on epidemiology, pathophisiology and risk factors for both entities, considering the differences and similarities that could be found in between them. We discuss traditional risk factors, obtained from epidemiological data, and also some novel ones, such as hyperhomocisteinemia or sleep apnea. We separate risk factors, as clasically, in two groups: nonmodifiables, which includes age, sex, or ethnicity, and modifiables, including hypertension, dyslipidemia or diabetis, in order to discuss the role of each factor in both ischemic events, ischemic stroke and coronary heart disease.

## 1. BACKGROUND

Ischemic heart disease and ischemic stroke are common entities that share in many cases a similar pathophysiology, based on arteriosclerosis. Usually, arteriosclerosis affects the patient widespread, so he becomes at risk for acute coronary syndrome (ACS) the same as for acute stroke. In both cases, a sudden change of circulation occurs, with resultant decreased blood supply to part of the heart or brain. Stroke, ocasionally, has been considered to be like a "heart attack" in the brain. Therefore it is clear that ACS and acute stroke share data on epidemiology, risk and etiological factors, and on therapeutic measures. Moreover, some studies have demonstrated that coronary artery disease is frequent among stroke patients, and also that chronic coronary artery disease also increases the risk of suffer a stroke [1-4].

However, heart and brain are two different organs in their anatomy, physiology and location, with their own circulation peculiarities. This fact explains that we find differences in risk factors, some being more likely to lead to an ACS than to a stroke, and viceversa. There are also notable differences in the etiology, much more diverse in stroke than in the ACS, and, consequently, in treatment. 

In this chapter we discuss the epidemiology and the risk factors for both diseases, analyzing their similarities and differences.

## 2. EPIDEMIOLOGY OF CARDIOVASCULAR DISEASES

Cardiovascular system diseases represent the leading cause of death worldwide, although the mortality for this cause is falling gradually due to advances in diagnosis and therapy. According to World Health Organization (WHO) data, in 2008, the mortality rate due to these diseases was 214-455 deaths per 100,000, being lower in developed countries [5].

If we look into the European Community, cardiovascular system diseases are also the leading cause of death in adulthood [6,7]. In 2005 the death rate from circulatory system diseases was 241.2 per 100,000 (295.4 in males, 196 in females). This rate decreased to 226.1 (273 and 183, respectively) the following year [8].

In Spain, there were 385,361 deaths in 2007. Analyzing the major groups of diseases, cardiovascular disease ranks first as cause of death in the year 2007, accounting for 32.2% of all deaths. For specific causes, ischemic heart diseases were the leading cause (37,222 deaths) and cerebrovascular disease the second (33,034 deaths) [9]. In Catalonia, also in 2007, cardiovascular diseases were the second leading cause of death in men younger than 85 years (after the tumors) and the first in men older than that age; in women, there were the first cause after 75 years old [7].

All these data give a clear idea of the extent and severity of these diseases. However, there are some differences in between the epidemiology of ischemic heart disease and cerebrovascular disease, which are outlined in the following paragraphs.

### 2.1. Epidemiology of Ischemic Heart Disease 

As discussed, coronary heart disease (CHD) is the leading cause of death in adulthood, accounting for 9.6% of deaths from specific causes. In Spain, in 2007, it was the leading cause of death among men (21.248, 10.6%) and the second leading cause of death among women (15.974, 8.7%) [9]. In the European Community, ischemic heart disease caused 89.1 deaths per 100,000 in 2006 (123.2 in males, 62.4 for females) [6].

The incidence of myocardial infarction (MI) varies according to the study and it varies also according to the different age and sex groups. The WHO MONICA project, where data of 37 populations were collected during a 10-year period, showed mean incidence of CHD events of 434 per 100.000 men-year [10]. Finland and United Kingdom (Belfast and Glasgow) had the highest rates (more than 700 per 100.000) and the lowest were in China (81 per 100.000) and Catalonia (210 per 100.000). In the same project, annual rates were quite lower in women: mean incidence rate was 103 events per 100.000 women-year. Incidence was higher in United Kingdom populations (188 and 265 per 100.000) and lower in China, Catalonia (35 per 100.000) and Toulouse (36 per 100.000). 

Several epidemiological studies in the U.S. have placed the incidence between 200 and 300 cases per 100.000 [11-17], being clearly higher in males (up to 486 cases per 100.000). This gender difference, which is found in all studies, appears to be more evident in younger ages of life, while at older ages tends to equalize. 

As discussed below, and unlike what happens with stroke, the incidence of ACS is highest in the 5th and 6th decade of life, to decrease later on [18]. 

The incidence of ACS follows a circadian rhythm, with a peak from 6:00 to noon when more than 1/3 of ACS occur, and a three-fold increase in the frequency of onset of MI at peak (9 am) as compared with trough (11 pm) periods [19,20]. In the study of Goldberg *et al.* [21], 23% of patients reported onset of symptoms within 1 hour after awakening. 

### 2.2. Epidemiology of Stroke

Cerebrovascular diseases are the second leading cause of death, also the second leading cause of dementia, and the leading cause of disability. By sex, they are the third leading cause of death in men, after ischemic heart disease and lung cancer, whereas in women they are the primary cause (18.964 female deaths from this cause in Spain in 2007, representing 10.2% of all deaths) [9]. In Catalonia, stroke causes 9.2% of overall mortality, 7.5% in males and 11.1% in women. The stroke mortality rates are clearly higher in non-industrialized countries [5]. 

The incidence of stroke varies widely depending on the study. The WHO places the global incidence of stroke at around 200 cases per 100.000 inhabitants [22], although data vary among countries. In a review of population-based studies since 1990, incidence varied from 130 to 410 cases per 100.000 person-years [23]. Highest rate was in Japan and lowest in United Kingdom, Germany and New Zealand. In the same review, the age-specific incidence of stroke increased progressively, and the range of stroke incidence in people aged 55 years or more was 420-650 per 100.000, except in Japan, Russia and Ukraine, where rates were higher. When difference by gender was analyzed, the incidence resulted higher in males in all studies.

Stroke, the same as myocardial infarction, also follows a circadian rhythm, and very similar to ischemic heart disease. To estimate the start time of the stroke is not easy, given that nearly a quarter of strokes occur during sleep [24]. Even taking this into account, a peak incidence has been observed between 6 am and 12 noon, when the 55% of ischemic strokes occur [25].

## 3. RISK FACTORS

An ischemic injury is a common mechanism for ischemic stroke (IS) and also for CHD. We find, logically, that many of the documented risk factors are present in both conditions, even if their contribution to the pathological event may be different. This fact is reviewed by Donnan *et al.* [26], who noted that identifiable risk factors explained 90% of CHD but only 60% of IS. Recently, an interesting paper has been published about this issue, in order to contrast risk factors pattern for each disease. It is a prospective cohort study conducted in Australia, on 2805 men and women, older than 60, over a 16 year follow-up period, where the authors identify factors which predict similarly IS and CHD, while other factors predict only one of each condition [27]. 

It has to be noted that, since CHD and IS share many risk factors and aspects of pathophysiology, to suffer IS and/or CHD will involve a risk of a new ischemic event. In patients with a history of CHD, the risk of repeating a new ACS is much higher than the risk of IS; and in individuals with a history of IS, the risk of recurrent stroke is higher than the risk of ACS [27]. However, stroke is a major complication associated with high mortality in patients with CHD, especially when hypertension is also associated [28]. On the other hand, the risk of CHD in patients with a history of IS or TIA is moderately high [2]. A necropsy study of patients with fatal stroke found atherosclerotic plaques in the coronary arteries in 72.4% of patients, stenosis greater than 50% in 37.5 % of them and myocardial infarction in 40.8% of the cases. These figures were roughly three times higher than what found in patients with other non-vascular neurological diseases [29].

Pathophysiology of ischemic cardiopathy and stroke have some similarities but also some differences. Both share atherothrombosis as an etiological factor, but the frequency with which this is the major mechanism of ischemia varies in each case. Thus, rupture or erosion of vulnerable plaques in coronary arteries “causing” severe stenosis or occlusion are the commonest cause of MI [30,31], while coronary spasm, emboli or dissection are less frequent etiologies, accounting for only 5-15% of acute MI. A similar proportion of patients have angiographically normal coronary arteries [32-35]. In the case of stroke a wide variety of etiologies may be found. In fact, stroke is not a simple disease but the manifestation of several diseases with different pathophysiology. There are ischemic strokes and hemorrhagic strokes, whith a different mechanism while sharing some risk factors. In this article we will only refer to IS, which itself has a heterogeneous etiology and pathophysiology.

TOAST classification [36] defines 5 types: large artery disease (atherothrombotic), cardioembolism, small vessel disease, non-habitual and cryptogenic. Frequency of atherotrombotic stroke is only 15-48% (much less than in CHD), a cardioembolic source is found in 21-37% cases, small-vessel disease is present in 10-34% and etiology is not found in as many as the 38% cases [37-42]. Given this greater variety of etiologies risk factors for the different subtypes of stroke will also vary, with each risk factor playing a different role in each subtype of stroke considered. Hereafter, the importance of different risk factors for both ischemic heart disease and ischemic stroke will be discussed. 

Vascular risk factors are usually divided into modifiable and non-modifiable. In this article we employ the same classification to review each factor, considering their role in cerebral and cardiovascular disease. These factors, together with a qualitative assessment of its influence in both patologies, based on its prevalence and its relevance, are summarized in Table **1**.

### 3.1. Non-Modifiable Risk Factors

#### 3.1.1. Age

Age is the strongest determinant of stroke, which is less common before 40 years old. According to data obtained from Framingham study, incidence of stroke increased steeply with age, becoming double in each successive decade from 55 years old on [43]. Hence, prevalence of stroke for individuals older than 80 years is approximately 27% compared with 13% for individuals 60-79 years of age [44]*. *The Framingham stroke risk profile (FSRP), developed decades ago, is still a useful tool to estimate, according to age, the specific 10 years probability of stroke by using clinical information, although conclusions should be drawn with caution. For instance, according to FSRP, the 10 years probability of stroke results 3% in a 60 years old man but 9,7% in men older than 84, independently of other risk factors [45]. 

In the case of CHD, age is also, by far, one of the most powerful risk factors. The highest incidence of ACS is found in younger patients than stroke, around the 5^th^ and 6^th^ decades of life [18, 46]. Even though, a quarter of ACS patients are younger than 55 years, and a similar proportion are older than 75. An observational study conducted in Spain showed that 36% of patients were younger than 45 years old [46]. 

#### 3.1.2. Sex

MI, the same as IS, is clearly more frequent among men. In Dubbo Study, male gender predicted similarly CHD and IS [27].

A recent meta-analysis [47] showed that stroke is 33% more incident in men than in women. This fact is also observed in the FSRP, mentioned above. However, due to longer life expectancy and much higher incidence of stroke in older ages, women suffer more strokes than men [48]. Etiology and risk factors of stroke are not the same in men than in women: cardioembolism is the main cause of stroke in women, whereas large and small vessel disease is the main cause among men [49]. Women use to suffer stroke earlier than men and their risk factors’ profile is different [50]. Turtzo *et al.* [51], when reviewing these different characteristics, point that more women than men die from stroke and that, in women, there is a trend for increased stroke severity, for a recurrent stroke after 5 years, and for a poorer functional outcome after IS.

Regarding CHD, its prevalence is also higher among men. The proportion of males rounds 64-71% in different series. This percentage increases in younger people and it tends to equalize when growing old [18, 52-54]. The most common found manifestation of CHD in women is angina pectoris, while in men is myocardial infarction [55].

#### 3.1.3. Ethnicity

Stroke incidence is greater among black patients, approximately double than among white patients according to several studies. The study conducted in Manhattan by Sacco *et al.* [56] found that stroke incidence among black patients was 2.4 times that of white patients, and that among Hispanic patients it was 1.6 times that of white patients. 

Different causes and mechanisms for stroke have been found to be more common in different race-ethnic groups, for example: hemorrhagic stroke –usually related to HBP- and young adults arteriopathy in Japan, extracranial atherosclerosis lesions in white patients vs. intracranial lesions in black, Hispanic and Asian patients. The reasons for that are still unclear. In the case of Africans an increased risk of ischemic stroke has been described related to sickle cell trait, but there is insufficient evidence to suggest an independent association [57].

The same as stroke, ischemic heart diseases may differ across ethnics groups. In the case of Hispanics, they have excessive rates of diabetes, obesity, dyslipidemia and metabolic syndrome, being particularly vulnerable to heart failure [58]. South Asian immigrants in the United States also have a higher risk for CHD, compared to Caucasians, and a higher prevalence of modifiable risk factors like diabetes or dyslipidemia. Nevertheless, the presence of risk factors may not be the only explanation for a high risk of ischemic events in this population, and new factors as dysfunctional HDL have been reported to play a role [59].

#### 3.1.4. Genetics-Heredity

Stroke incidence increases when there is a family history of stroke. The same occurs with CHD disease incidence, as observed in The Framingham Study: a family history of ischemic heart disease in first degree relatives younger than 55 implies a relative risk of 1,5-1,7, independently to other risk factors [44]. These facts could be due to a familial association existing with other risk factors for the disease (cholesterol, hiperfibrinogenemia, HBP, diabetes…..), or due to independent factors, such as the controversially investigated adrenergic receptor gene. Rexrode *et al.* [60] did not find association between the adrenergic receptor genetic variation studied and the risk of cardiovascular disease –MI or IS- in women. A recent study by Schurks *et al.* [61] suggested that the Haplotype Gly16-Gln27-Thr164 polymorphism in the beta2-adrenergic receptor which had previously shown a protective effect on MI in men, is also associated with reduced risk of MI in Caucasian women, but not with an IS risk reduction. 

Previously, an increased risk for both CHD and IS had been demonstrated in carriers of e4 allele for Apolipoproteine E. In young patients, several hereditary arteriopathies might be found to be the cause of ischemic stroke, such as CADASIL, CARASIL, Sneddon syndrome, mitocondrial cytopathies, Fabry disease or different coagulopathies. All of them are rare in the case of stroke, and even rarer as a cause of CHD. 

#### 3.1.5. Uncommon Related Factors

Many other factors have been said to affect the epidemiology of IS and/or CHD, although mechanisms are not well clarified and data are still lacking about it. Just to mention, 

-Size: it has been said to be inversely associated with the long-term incidence of fatal stroke; in contrast, height is not significantly related with mortality due to CHD mortality [62].

-Body fat distribution: it predicts long term stroke mortality and also long term heart disease mortality in middle-aged men [63].

-Year station: a higher incidence of IS and of acute MI has been described related to changes –descent- in atmospheric pressure [64] and during winter [65].

### 3.2. Modifiable Risk Factors

In this section we discuss modifiable, or treatable, risk factors for CHD and IS such as living habits, some atherogenic personal attributes (HBP, DM, DLP, obesity…) and certain diseases, which predispose to the occurrence of an ischemic event. Identified modifiable risk factors are responsible for 90% of myocardial infarction [66] while they explain only about 60% of strokes [26].

These risk factors may have changed over the last years, as observed in the Lausanne Stroke Registry [67] where an increasing proportion of hypercholesterolemia but a decreasing proportion of high blood pressure, smoking habit and diabetes mellitus in patients were described over time.

#### 3.2.1. High Blood Pressure

High blood pressure (HBP) or hypertension affects nearly 30% of the world’s population. It is a risk factor for stroke, CHD, chronic heart failure, kidney failure, vascular dementia and, in general, for all cardiovascular diseases. According to WHO data, 62% of all strokes and 49% of CHD are attributable to HBP.

The Framingham Heart Study [68] showed an increased relative risk (RR) of cardiovascular disease of 2, in patients with blood pressure 130-139/ 85-89 compared with patients with blood pressure lower than 120/80. The risk of CHD and stroke increases linearly when blood pressure is higher than 115/75. Mortality due to IS and to MI also increases linearly and progressively when blood pressure is above 115/75, being double for every 20mm Hg systolic or 10mm Hg diastolic increase in blood pressure [69].

##### Blood Pressure and Stroke

HBP is the most important modifiable risk factor for a stroke of any type, ischemic or hemorrhagic, and also for transient ischemic attack. HBP accelerates the progression of atherosclerosis, one of the main predictors of ischemic stroke. In Framingham Study, most hypertension related strokes were due to atherothrombotic brain infarction. Thus, hypertension is a main risk factor not only for hemorraghic stroke but also, and at a similar extent, it is a main risk factor for IS [70]. Systolic, diastolic or both blood pressure values can contribute to the occurrence of stroke when they are high. In large-scale prospective cohort studies it has been observed that relative elevations of blood pressure, often undervalued by patient and doctors, may also increase the risk: 140-160/90-94 increases the risk of stroke by 1.5, >160/95 increases the risk by 3-4. Decreasing blood pressure, even if modestly, may achieve 40% risk reduction of stroke, as described in prospective clinical assays [71,72]*. *

When analyzing series of patients affected of ischemic stroke, HBP is found in 53-68% of patients [37-39]. The contribution of HBP to the occurrence of each different subtype of stroke is not the same, as reported in these series. In the Athens Stroke Registry [39], HBP is present in 80% of lacunar infarcts, in 73% of atherothrombotic, in 62% of cardioembolic, and in 62% of cryptogenic strokes. The Barcelona Stroke Registry [38] showed a predominant role of HBP in lacunar infarction, as well (76%). 

A recent meta-analysis [73] showed the relation between higher diet salt intake and greater incidence of stroke (1.23 relative risk). In the Dubbo study [27] HBP appears to be a stronger predictor of IS, compared to CHD. 

##### Blood Pressure and CHD

HBP is one of the main risk factors for CHD. According to the GRACE score, systolic blood pressure is the modifiable factor which better predicts acute myocardial infarction and related death [66]. Epidemiological data and other epidemiological studies have demonstrated that the incidence of cardiovascular diseases increases incrementally with blood pressure, even within the normal range (45% of cardiovascular events reported at Framingham study occurred at a systolic BP of 140 or lower). Although a threshold for the association between BP and cardiovascular risk is not well established, normal and high-normal BP conferred almost a 2-fold increased risk factor for cardiovascular disease, compared to optimal BP. These studies, as reviewed by Kannel *et al.* [70], also showed that the combination of other risk factors such as metabolic syndrome greatly augments the hazard ratio of elevated blood pressure. 

The study by Strazzullo *et al.* [73], already mentioned, shows a greater incidence of cardiovascular disease related to a higher salt intake (1.14 relative risk).

#### 3.2.2. Diabetes Mellitus

Diabetes mellitus (DM) is a disease with increasing prevalence [74]. It is associated with a high risk of both ischemic cardiopathy and cerebrovascular disease, for being a clearly predisposing factor for the development of both large-vessel and small-vessel atheroma. Diabetes pathophysiological mechanisms to cause vascular disease are multiple [75]. It is estimated that 44-52% of deaths in diabetic patients are due to cardiovascular diseases [76]. Results are different depending on when the diagnosis of diabetes is established, before or after age 30. A study carried out in Wisconsin showed that, among patients with a younger onset of diabetes, ischemic cardiopathy and cerebrovascular disease caused 26.9% of deaths (the more frequent cause of death in this group is diabetes) while in patients with older onset this rate was 47.5% [77]. As discussed below, these deaths are mainly due to CHD.

##### Diabetes Mellitus and CHD

DM is a key-factor in the occurrence of coronary syndrome. The prevalence of DM in ACS varies between 21.1 and 31.7% among registries [52-54]. In the Honolulu Heart Program [78], a prospective cohort study which followed more than 7,000 men for 23 years, the age-adjusted rate of CHD was 14.1 per 1000 person-years in men with known diabetes, 8.8 in those with asymptomatic hyperglicemia and 5-5.9 in groups with normal glucose. This risk is not limited to men, as diabetic women have been also found to have an increased risk of CHD compared to men [55]. As noted earlier, ischemic heart disease is a major cause of death in diabetic individuals. In the study by Moss *et al.* [77], DM was the cause of death in 24.8% of people with younger onset DM and in 38% of individuals with older onset DM. 

##### Diabetes Mellitus and Stroke

DM is also a risk factor for the occurrence of ischemic stroke. In the Honolulu Heart Program, subjects with known diabetes and asymptomatic hyperglicemia showed an increased risk of ischemic stroke, and these associations were independent of age and other vascular risk factors [79]. Moreover, a recent study showed that in patients with a previous TIA or previous minor stroke, glucose intolerance increased the risk of stroke [80]. However, not all ischemic strokes are equally favoured by the DM: prevalence of DM is 18-32% in atherothrombotic infarction, 20-32% in lacunar infarction and somewhat less in cardioembolic one (8-21%), depending on the series [37-39,81]. 

In diabetic patients, cerebrovascular disease is a far less common cause of death than coronary heart disease. In the study by Moss *et* *al*. [77], cited previously, the stroke was the cause of death in 2.1 of younger onset diabetic patients and in 9.5 of the older onset group. 

#### 3.2.3. Dyslipidemia

Hypercholesterolemia is a factor that has been demonstrated clearly related to atheromatosis [82]. Several studies demonstrate a direct relationship between cholesterol levels, triglyceride levels and cardiovascular mortality [83,84]. Even if dyslipidemia has been considered -and is still being considered- a risk factor for CHD and also for stroke, it may have a different relevance in each entity. At the moment no one discusses the relationship between high values of cholesterol and LDL-cholesterol, low levels of HDL-cholesterol and the presence of coronary atheroma and symptomatic coronary disease. However, the relationship between cholesterol and stroke appears to be more controversial and inconsistent across studies, probably because stroke is a much more heterogeneous disease. Many series, especially the older ones, did not differ neither ischemic from hemorrhagic strokes in their analysis, nor the different types of ischemic stroke depending on etiology. Different data about the relationship between hypercholesterolemia and the two entities are exposed below. 

##### Dyslipidemia and CHD

The relationship established for dyslipidemia and the development of atheroma implies a close relationship with the development of CHD, as well. A high LDL and/or low HDL-cholesterol levels predict coronary events [27,85]. In the Asia Pacific Cohort Studies Collaboration (APCSC) study, each 1-mmol / l higher level of total cholesterol was associated with 35% increased risk of coronary death [86]. Different registers of CHD showed a proportion of patients with dyslipidemia of 45-54%, which is higher than the proportion of patients with diabetes [52-54]. 

##### Dyslipidemia and Stroke

As discussed earlier, hypercholesterolemia has not been clearly established as a risk factor for stroke. There are some studies which found no significant relationship between cholesterol levels and incidence of stroke [87,88], even when analyzing hemorrhagic and ischemic stroke separately. By contrast, in the APCSC study each 1-mmol / l higher level of total cholesterol was associated with 25% increased risk of ischemic stroke (less than increased risk seen in the case of CHD, but still a significant risk), and with 20% decreased risk of fatal stroke hemorrhagic [86]. In the study by Simons *et al.* [27], unlike what observed with CHD, a high LDL-cholesterol level predicted IS, but HDL- cholesterol level did not. This lack of consistency has been suggested to be due to the heterogeneity in the pathogenesis of ischemic stroke, thus some subtypes of stroke would be more related to hypercholesterolemia [89]. This fact is reflected in prospective registries of stroke, where dyslipidemia is described in up to 46% of atherothrombotic infarctions and up to 38% of lacunar infarctions, but in less than 20% of cardioembolic ones [37-39].

#### 3.2.4 Smoking

Relationship in between smoking habit and athero-sclerosis has been clearly established. Tobacco contributes to the progression of the atherosclerotic plaque and to its instability. It facilitates platelet aggregation, increases blood viscosity and damages the endothelium, among other mechanisms of action [90-94]. So, increased risk of CHD and IS occurs in smokers, as demonstrated, almost unanimously, in many studies. 

##### Smoking and CHD

Tobacco use is strongly associated with coronary disease. Studies have also shown that the risk increases with the number of cigarettes [95-97]. In a cohort of nearly 120.000 female nurses, among smokers of more than 25 cigarettes per day, the relative risk of suffering from fatal CHD was 5.5 [95]. In the Copenhagen City Heart Study, the risk increased 2-3% for each gram of tobacco smoked daily [96]. In any case, even smokers of 1-4 cigarettes per day have increased risk of acute myocardial infartion [95]. Some studies conclude that the association of tobacco with CHD is higher in women than in men [96] and that this risk is also increased when smoking habit is associated with the use of oral contraceptives [98]. 

The attitude of quitting the habit is the best preventive measure, as ex-smokers can see their risk of CHD decrease over the years [95,96,99], reaching a risk similar to a non-smoker person (someone who has never smoked) after about 10 years. 

##### Smoking and Stroke

Smoking is an independent risk factor for all subtypes of ischemic stroke. A meta-analysis concluded that the relative risk of cerebral infarct associated with cigarette smoking was 1.9 [100], with differences depending on age groups: RR in < 55 (before 55 years old) was 2.9, in age 55-74 was 1.8 and in patients older than 74, 1.1. As in CHD, risk was higher in women than in men and the risk was dose-dependent. In an epidemiologic study conducted in Minnesota, the relative risk resulted 3.6 in patients aged 50, 1.9 in patients aged 70 and non-significant after 80 years old [101]. Risk of stroke was about twice as high for passive smokers than for non-smokers [102,103]. 

Ex-smokers may reduce their risk of suffering a stroke till a RR of 1.2, although this RR remains higher in older people [100]. In the study conducted by Whisnant *et al.* [101], the increased risk was higher when comparing current smoker vs never & past smokers than when comparing people who ever smoked vs never non-smokers.

#### 3.2.5. Alcohol

Alcohol, taken in moderation, has proven to be a protective factor for cardiovascular disease, especially for CHD. Several hypotheses have been postulated to explain this fact, related to the effects of alcohol on lipid levels and platelet aggregation [104,105], to an effect on increased levels of estrogen [106], or even related to the assumption that moderate alcohol drinking is synonymous with social integration [107]. However, this beneficial effect only occurs for moderate alcohol intake, because, as discussed below, alcohol abuse is harmful and increases the risk of CHD, the same as stroke.

##### Alcohol and CHD

Prospective studies have reported a lower risk of death from CHD among moderate drinkers, compared with nondrinkers [108-111]. In a prospective study conducted in Denmark, men who drank daily a mean of 12 g of ethanol had the lowest risk to develope CHD [111], even less than men who drank less than once a day. In women, risk was also reduced if they drank at least one day a week, but no difference was found between drinking frequency. 

However, it has to be remarked that binge drinking increases the risk of suffering CHD. In heavy drinkers, RR of death from cardiovascular disease was 2.05, most of them due to myocardial infarction [112]. Hence, adults’ risk of CHD in relation to volume of alcohol is described as a U-shaped curve [110].

##### Alcohol and Stroke

As we have seen with ischemic heart disease, it seems to be a dual relationship between alcohol consumption and incidence of ischemic stroke. Moderate alcohol consumption is associated with lower incidence of ischemic stroke [113,114], but, on the other hand, heavy drinkers have a higher risk of stroke [115]. It has been suggested that recent alcohol consumption may act as trigger [116]. 

#### 3.2.6. Obesity 

Obesity is clearly associated to cardiovascular disease, IS and CHD. Firstly, a higher proportion of HBP, DM and dyslipidemia –three key factors related to cardiovascular risk- can be found among patients with obesity [117, 118]. Moreover, these patients use to have a more sedentary live and a less healthy diet than patients without obesity. 

In a recent meta-analysis [119] the incidence of each co-morbidity related to obesity and overweight has been estimated. Among these co-morbidities risk were estimated for CHD and stroke. For men, RR for CHD was 1.81 for obesity based on waist circumference (WC) and 1.72 for body mass index (BMC). Estimated RR for women were higher: 2.69 for obesity based on WC and 3.10 for BMI. Obesity also increases risk of stroke but RR was lower than for CHD. RR was 1.51 for males and 1.49 for females. The heterogenicity present in stroke phisiopathology may explain this risk, lower for IS than for CHD. 

Taking into account that obesity is an increasing health problem, it is estimated that the prevalence of CHD will increase by a range of 5 to 16% due to this factor [120].

#### 3.2.7. Physical Inactivity

Physical activity reduces risk of both CHD and IS. The reason for this beneficial effect is multifactorial. First, regular exercise lowers blood pressure and improves lipid profiles [121]. Physical activity also improves endothelial function [122,123]. Other possible mechanisms, such as the reduction of blood viscosity, fibrinogen levels, and platelet aggregability may contribute to the reduction of cardiac and cerebral ischemic events [124].

##### Physical Inactivity and CHD

Many studies have demonstrated that a sedentary life increases risk of CHD. A meta-analysis estimated a RR of death from CHD of 1.9 in people with sedentary occupations [125]. In a prospective study with post-menopausal women, both walking and vigorous exercise were associated with significant reduction in the incidence of CHD (closer to 50%), irrespective of their age, race or body-mass index [126]. In patients with previous angina, regular exercise reduces the incidence of recurrences [127].

##### Physical Inactivity and Stroke

Similar to what is observed in ischemic heart disease, moderate and high levels of physical activity reduces the risk of IS. Compared to low-active individuals, moderately active individuals have a 20% lower risk and highly active individuals had a 27% lower risk of stroke incidence or mortality [124]. This decreased risk is related to both intensity and duration of activity [128].

#### 3.2.8. Diet

Mediterranean diet is characterized by a high intake of monounsaturated fat, plant proteins, whole grains and fish, while a low consumption of red meat, refined grains, and sweets [129]. 

It is associated with beneficial effects on lipids, blood pressure and also inflammatory markers. Several studies have documented an inverse relationship between the adherence to the Mediterranean diet and the incidence of CHD and stroke. 

##### Diet and CHD 

There are many studies, in many different populations, concluding that the Mediterranean diet is protective against the incidence of CHD. The Lyon Diet Heart Study, conducted in patients with previous MI, showed that the Mediterranean diet prevents the incidence of both cardiovascular and, specifically, coronary events [130]. Similar results were found in the HALE project, a study conducted in several european countries, including elderly people (hazard ratio: 0.61) [131]. CHD mortality was also reduced in elderly people with a greater adherence to Mediterranean diet (adjusted hazard ratio, 0.67). In a recent study including women aged 38-63, women with a higher adherence to Mediterranean diet had a lower incidence of CHD [132]. 

##### Diet and Stroke 

Only a few studies have specifically evaluated the effect of Mediterranean diet on the incidence of ischemic stroke. In the Nurses' Health Study, discussed earlier, women who adhered to the Mediterranean diet more closely had also a lower risk of IS [132]. In a previous study by Joshipura et al., it had been shown that the consumption of fruit and vegetables, basic part of the Mediterranean diet, significantly reduced the incidence of IS [133]. 

#### 3.2.9. Atrial Fibrillation and other Sources of Embolism

Cardioembolism is the second most common cause of ischemic stroke, accounting for 21-38% of ischemic strokes [37-39]. In contrast, coronary embolism is a rare cause of MI. Sequelae from a MI, as a ventricular segmental akinesia, ventricular aneurysm or ischemic cardiomyopathy may become an embolic source and therefore be the cause of cardioembolic brain infarction.

Atrial fibrillation (AF) is the most common cardiac source of embolic stroke, accounting for up to 50% of patients. This proportion increases with age. Other common causes of cardioembolic stroke are myocardial infarction (30%), left ventricular thrombus, valve disease or prosthetic valves [134].

Risk of ischemic stroke among patients with AF averages 3-4% per year, but it varies widely depending on several factors [135,136]. In a sistematic review of the literature, 4 consistent independent risk factors for stroke in patients with atrial fibrillation were found: prior stroke, increasing age, previous hypertension and previous diabetes. Prior stroke or TIA was the most powerful of these risk factors and confered a high stroke risk (average 10% per year) [135]. Age was also a consistent independent predictor of stroke in patients with AF, with an incremental increased risk of 1.5 per decade [135]. 

AF could also be considered a risk factor for CHD. In the Dubbo Study [27], AF predicted CHD, but it was a higher predictor for IS than for CHD. In fact, AF was, statistically, the most powerful predictive factor for IS. 

#### 3.2.10 Other Risk Factors

##### Hyperhomocysteinemia

It has been shown that homocysteine causes changes in the arterial wall, by different mechanisms, implying an increased risk of ACS and IS in subjects with hyperhomocysteinemia. This fact has been reflected in numerous studies. 

In a meta-analysis performed by Ford *et al*. [137], each increase of 5 umol / l in homocysteine concentration corresponded to a non-significant OR for CHD of 1.06 for 2 publications of cohort studies, 1.23 (95% CI 1.07-1.41) for nested case-controls studies and 1.70 (1.50-1.93) for case-control studies. Similarly, the same trend is observed for cerebrovascular disease. The summary OR for a 5-umol / l increase in homocysteine concentration were 1.10 (0.94-1.28) for cohort studies, 1.58 (1.35-1.85) for nested case-control studies and 2.16 (1.65-2.82) for case-control studies. Hyperhomocysteinemia has been associated with both carotid and intracranial circulation disease, with a lower development of collateral circulation, and also with other risk factors directly related to CHD and to cerebrovascular disease, like hypertension [138]. 

##### Hypercoagulopathy 

Inherited thrombophilia such as deficiency of antithrombin III, protein C or protein S, mutation of prothrombin gene G20210A or factor V Leiden have been associated to venous thrombosis, but their association with arterial disease remains controversial. Presence of an antiphospholipid anti-body, such as lupic anticoagulant or anticardiolipin antibody may also lead to hypercoagulopathy. Different studies failed to find an etiological association between these factors and coronary disease or ischemic stroke, but some trials have suggested that thrombophilia should be important as an etiological factor in young subjects [139-141].

##### Lipoproteins 

Lipoprotein (a) is an atherogenic lipoprotein which has been postulated as an independent risk factor for cardiovascular disease. Its plasma levels are not determined routinely. High levels of lipoprotein (a) have been correlated with both CHD [142] and IS [143], although their etiologic association is not clear. In the ARIC study, high levels of lipoprotein (a) significantly correlated with higher incidence of IS in blacks and white women, but not in white men [144]. ApoB/ApoA1 ratio also was associated with myocardial infarction in the INTERHEART study [145]. In the Dubbo Study, both parameters were only predictors of an increased incidence of CHD, but not of IS [27]. 

##### Inflammatory Markers 

Since atherosclerosis is a chronic inflammatory process, several markers of chronic inflammation have been studied as potential cardiovascular risk factors. C-reactive protein level is a strong predictor of cardiovascular events, even more than LDL cholesterol [146], and for both CHD and IS [147-150]. Elevated leukocyte count is associated with coronary events, but in a recent prospective cohort study it was not associated with incidence of stroke [151]. 

##### Obstructive Sleep-Apnea Syndrome (OSAS)

Many studies have shown that sleep disorders, especially OSAS, are associated with increased cardiovascular risk. Individuals with these disorders have 2-4 times greater risk of suffering an ischemic stroke [152]. A similar risk for both ACS and IS have been described in these patients [153]. Several pathophysiological mechanisms have been postulated to explain this increased risk, such as the development of hemodynamic changes, atherosclerosis, arrhythmias, endothelial dysfunction and prothrombotic states, among others, all of them facilitated or triggered by hypoxemia and / or apnea / snoring [152]. 

## 4. SUMMARY

In summary, CHD and cerebrovascular disease share similar pathophysiological mechanisms and, consequently, many risk factors. However, the more complex etiology demonstrated for IS lead to a different incidence of these factors in each disease, CHD and IS, the same as in every stroke subtype. 

## Figures and Tables

**Table 1:** Vascular Risk Factors: Relevance in Ischemic Cardiopathy and Ischemic Stroke

	Ischemic Cardiopathy	Ischemic Stroke
**Non-modifiable:**		
• Age	+++ (50-70 y.)	+++ (older)
• Sex	++ (male)	++ (male)
• Ethnicity	+	+
• Genetics-heredity	++	++
• Uncommon related factors	+	+
**Modifiable:**		
• High blood pressure	+++	+++
• Diabetes	+++	++ (aterothrombotic-lacunar)
• Dyslipidemia	+++	++ (aterothrombotic-lacunar)
• Smoking	+++	+++
• Alcohol	+ (dual relationship)	+ (dual relationship)
• Obesity	+++	++
• Physical inactivity	++	++
• Diet	++	++
• Atrial fibrillation and other sources of embolism	+	++ (cardioembolic)
• Other risk factors:		
-Hyperhomocysteinemia	++	++
-Hypercoagulability	+	+
-Lipoproteins	++	+
-Inflammatory markers	+	+
-Obstructive sleep-apnea syndrome	++	++
